# A Case of GATA3 Positive Pleomorphic Liposarcoma, Epithelioid Variant: A Diagnostic Pitfall

**DOI:** 10.1155/2023/9443027

**Published:** 2023-03-24

**Authors:** Makoto Abe, Nobuo Hoshi, Sayuri Hoshi, Kaoru Hirabayashi, Kazutaka Kikuta, Toru Hirozane, Rumi Nakagawa, Tsukasa Mizuno, Hiroshi Nakamura, Koichi Inoue, Takehiko Yamaguchi

**Affiliations:** ^1^Department of Pathology, Tochigi Cancer Center, 4-9-13, Yohnan, Utsunomiya, 320-0834 Tochigi, Japan; ^2^Department of Musculoskeletal Oncology and Orthopaedic Surgery, Tochigi Cancer Center, 4-9-13, Yohnan, Utsunomiya, 320-0834 Tochigi, Japan; ^3^Department of Radiation Oncology and Image-Applied Therapy, Tochigi Cancer Center, 4-9-13, Yohnan, Utsunomiya, 320-0834 Tochigi, Japan; ^4^Department of Pathology, Nikko Medical Center, Dokkyo Medical University, 145-1 Moritomo, Nikko, 321-1298 Tochigi, Japan

## Abstract

Pleomorphic liposarcoma is a rare malignant adipocytic tumor showing undifferentiated pleomorphic sarcoma morphology with various degrees of epithelioid features. It is sometimes difficult to distinguish from carcinoma metastasis. Immunohistochemical panel is very important for differential diagnosis; however, there is a risk that unexpected staining could lead to misinterpretation. We report a pleomorphic liposarcoma, epithelioid variant, in an 88-year-old man, with tricky-positive staining for GATA3. Histological examination revealed a tumor with epithelioid morphology. The tumor consists of solid sheets of epithelioid tumor cells with focal aggregates of pleomorphic lipoblasts. Immunohistochemically, the adipocytic tumor cell areas were positive for S100 protein, and the epithelioid tumor cells showed CAM 5.2 positivity. GATA3 was diffusely positive. The combination of CAM 5.2 and GATA3 staining suggested the possibility of metastatic cancer, but systemic clinical examinations did not detect any presence of a primary tumor, including urinary bladder, breasts, and salivary glands. The pathological diagnosis of pleomorphic liposarcoma, epithelioid variant, was made because of the presence of malignant lipoblasts. Our report may contribute for differential diagnosis of pleomorphic liposarcoma, epithelioid variant, with unexpected positive immunoreaction for GATA3.

## 1. Introduction

Immunohistochemical staining is widely used and the important tools supporting morphological diagnosis. Unexpected staining could lead to misinterpretation; therefore, pathologists should recognize immunohistochemical staining, including aberrant expression and nonspecific staining, as a pitfall. Here, we report a pleomorphic liposarcoma, epithelioid variant, in an 88-year-old man, with tricky-positive staining for GATA3, a commonly used marker of urothelial and breast-specific origin. Importantly, there are limited cases of GATA3 expression in pleomorphic liposarcomas.

## 2. Material and Methods

The specimen was formalin-fixed and paraffin-embedded (FFPE) and stained using hematoxylin and eosin. Immunohistochemistry (IHC) evaluation was performed with an automated immunostainer (BenchMark Ultra, Ventana Roche Diagnostics) as per the manufacturer's instructions using the following primary antibodies: cytokeratin (CK) AE1/AE3 (clone AE1/AE3, 1 : 10, DAKO), CAM 5.2 (clone CAM 5.2, prediluted, BD Biosciences), GATA3 (L50-823, prediluted, NICHIREI), S100 protein (polyclonal, 1 : 500, DAKO), CD34 (NU-4A1, prediluted, NICHIREI), HMB45 (clone HMB45, 1 : 200, DAKO), Melan A (A103, 1 : 25, DAKO), MDM2 (IF2, 1 : 25, Life Technologies), CDK4 (DCS-31, 1 : 30, Invitrogen), p63 (4A4, prediluted, NICHIREI), CK7 (OV-TL 12/30, 1 : 25, DAKO), CK20 (Ks20.8, 1 : 25, DAKO), CDX-2 (DAK-CDX2, prediluted, DAKO), and TTF-1 (SPT24, prediluted, DAKO).

MDM2 FISH analysis using MDM2/CEP12 probe (J17911, Jokoh) to the MDM2 locus (12q15) and the chromosome 12 centromere (CEP12) is designed for formalin-fixed and paraffin-embedded tissue. FISH is performed on a tissue section considered a representative area, using manufacture's protocol. One hundred cells were evaluated with each probe and considered positive with at least 15 MDM2 signals per cell [1].

## 3. Case Presentation

**Figure 1:**
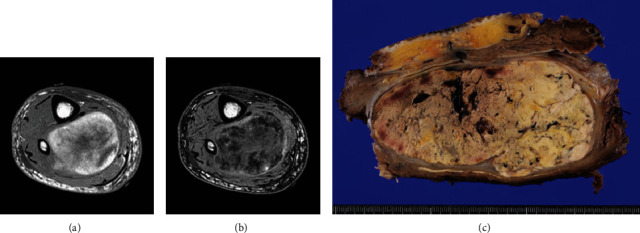
In-phase (a) and out-of-phase (b) of T1-weighted (W) magnetic resonance imaging (MRI). A 12.5 × 6 × 6 cm soft tissue tumor was identified in the soleus muscle of an 88-year-old man. A relatively well-defined intramuscular mass reveals a signal drop on out-of-phase T1-WI compared to in-phase T1-WI, indicating the presence of fat components. (c) Gross findings of surgically excised specimen. Cut surfaces of an intramuscular tumor are gray tan in color and show extensive areas of geographic necrosis with foci of hemorrhage.

**Figure 2:**
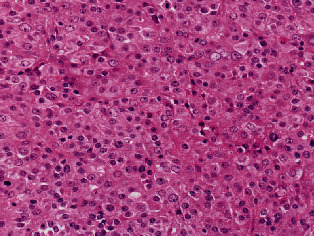
Histological findings of the biopsy specimen. Magnification: ×20. Epithelioid tumor cells are arranged in solid, cohesive sheets. Tumor cells exhibit round to oval nuclei with a relatively distinct nucleolus and abundant eosinophilic cytoplasm. These histological features are reminiscent of poorly differentiated carcinoma.

An 88-year-old man presented with swelling in the right lower leg. He had a history of chondrosarcoma of the rib and ascending colon cancer. There was no evidence of family history including Li-Fraumeni syndrome. Routine laboratory test results were unremarkable. Magnetic resonance imaging revealed a 12.5 × 6 × 6 cm mass in the soleus muscle of the right lower leg, which showed heterogeneous signals suggestive of necrosis and hemorrhage (Figures 1(a) and 1(b)). The border was well demarcated but partly obscure. Computed tomography (CT) did not reveal metastasis to other organs. The biopsy specimen showed solid growth of epithelioid tumor cells with enlarged round or oval nuclei and abundant eosinophilic cytoplasm (Figure 2). Numerous mitotic figures were seen. The tumor had well-developed capillaries and foci of geographic necrosis. No specific tumor differentiation was observed. The tumor was completely excised because of its malignant features.

**Figure 3:**
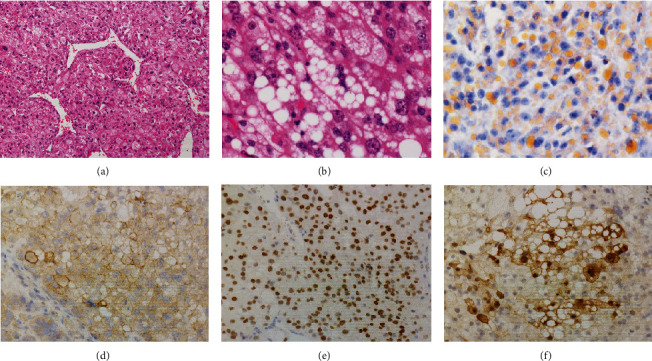
Histological findings of the surgical specimen. Magnification: (a, c–f) ×20, HE, (b) ×40. (a) An epithelioid tumor cell has well-developed staghorn-like (hemangiopericytoma-like) vasculature. (b) Pleomorphic multivacuolated lipoblasts are occasionally found. (c) Sudan III stain demonstrates fat droplets in the cytoplasm. (d) CAM 5.2 is focally expressed in epithelioid tumor cells. (e) The tumor nuclei are diffusely positive for GATA3. (f) S100 proteins are focally expressed in lipogenic tumor cells.

Grossly, the tumor showed a solid gray tan-cut surface, nodular growth pattern, and central necrosis (Figure 1(c)). Histologically, most areas consisted of sarcomas with epithelioid morphology, as observed in the biopsy specimen (Figure 3(a)). In other areas, the tumor cells had a clear cytoplasm, some of which demonstrated multivacuolated cytoplasm, i.e., pleomorphic lipoblasts (Figures 3(b) and 3(c)). Geographic necrosis and vascular invasion were observed. The myxoid matrix was not evident. The epithelioid areas were focally positive for CAM 5.2 (Figure 3(d)) and diffusely and strongly positive for GATA3 (Figure 3(e)). Lipogenic areas showed the expression of the S100 protein (Figure 3(f)). The tumor cells were negative for CD34, CDK4, HMB45, Melan A, p63, CK7, CK20, CDX-2, and TTF-1. MDM2 was negative in both immunohistochemistry and fluorescence in situ hybridization (not shown). Based on the overall morphological, immunohistochemical, and molecular findings, we diagnosed the tumor as a pleomorphic liposarcoma, epithelioid variant.

The patient developed multiple lung, liver, and bone metastases three months after surgery. The patient did not undergo any further treatment because of his age.

## 4. Discussion

Based on their clinical presentation and genetic background, liposarcomas are classified into six subtypes according to the current WHO Classification of Tumours, Soft Tissue and Bone Tumours (5th ed.) [2]. Pleomorphic liposarcomas are a rare subtype, pleomorphic sarcomas that show at least focal adipose differentiation; however, their genetic profile remains unclear [2]. They often have gene mutation of p53 and NF1, but no amplification of chromosome 12q14 (including the MDM2 gene) is found in atypical lipomatous tumor/well-differentiated liposarcoma and dedifferentiated liposarcoma, and no FUS-DDIT3 rearrangement is seen in myxoid liposarcoma [3, 4]. Tumors typically occur in adults over the age of 50 years, with a slight male predominance. Most tumors arise in the deep soft tissues of the thigh (75% of cases) and trunk [2]. Local recurrence and metastatic rates are 30–50%, with an overall 5-year survival rate of 50–60% [2]. Pulmonary and pleural metastases are common [5]. Diagnosis requires the presence of lipoblasts and other lipid droplets to confirm adipose differentiation. Most cases contain areas of myxoid stroma associated with pleomorphic lipoblasts. In approximately one-quarter of cases, the tumors also show varying degrees of epithelioid morphology [6, 7]. It is challenging to diagnose in the case of epithelioid morphology with inconspicuous lipid droplets resembling poorly differentiated carcinoma [7]. In such cases, an immunohistochemical analysis should be performed to rule out the possibility of metastatic cancer.

In this study, we have identified two important pathological issues. First, pleomorphic liposarcoma, epithelioid variant, expresses GATA3. GATA3 is a zinc finger transcription factor associated with cell development and differentiation. GATA3 is known to play an important role in regulating genes involved in mammary-gland morphogenesis and luminal-cell differentiation, epidermal and follicular morphogenesis, and endothelial cells, especially in large vessels [8]. Immunohistochemical nuclear staining for GATA3 in tumors is highly restricted to carcinomas of the breast, salivary gland, urothelial, and squamous epithelial origin [8]. Especially among breast cancer, GATA3 has diagnostic utility in triple-negative breast carcinomas (43%), typically negative for other mammary markers [9]. Expression of GATA3 in nonepithelial tumors is limited and is considered diagnostically useful only for paraganglioma and malignant mesothelioma (sarcomatoid type) [10–12]. Only a few cases of aggressive sarcomas, such as dedifferentiated chondrosarcoma or angiosarcoma, leiomyosarcoma, and malignant melanoma, have been reported with GATA3 expression [8, 13, 14]. To the best of our knowledge, GATA3 expression in pleomorphic liposarcomas has not been previously reported. Haraguchi et al. reported GATA3 expression in other subtypes of liposarcoma––18.1% (2/11) of myxoid liposarcoma cases, 42.9% (3/7) of well-differentiated liposarcoma cases, and 50.0% (1/2) of dedifferentiated liposarcoma cases [15]. Furthermore, they reported that GATA3 expression in soft tissue sarcomas is a poor prognostic factor [15]. The second pathological issue is the combination of cytokeratin and GATA3 expression that mimics metastatic carcinoma, especially urothelial carcinoma. Pleomorphic liposarcoma, epithelioid variant, may often show focal immunostaining with antibodies against cytokeratin (reported in about 50% of cases) [6]. In cases with predominantly epithelioid morphology and inconspicuous lipid droplets, as observed in this case, it is difficult to distinguish from metastases of poorly differentiated carcinomas, such as urothelial carcinoma, renal cell carcinoma, adrenocortical carcinoma, or other epithelioid subtypes of sarcoma, such as epithelioid sarcoma, malignant melanoma, and perivascular epithelioid cell tumor [5–7, 16]. Miettinen and Enzinger reported a case of nephrectomy mistakenly performed following a diagnosis of metastatic renal cell carcinoma [6]. Special staining such as orcein stain and Sudan stain is performed to prove adipogenic differentiation. Although a few immunostaining markers indicate adipogenic differentiation, the widely used S100 has low specificity [17]. Pleomorphic liposarcoma shows focal immunostaining with antibodies against cytokeratin, EMA, CD34, smooth muscle actin, and S100 [7]. Focal positivity for cytokeratins makes it challenging to distinguish carcinoma metastasis [7], especially when the patient has a history of carcinoma or other probable sites detected in imaging studies. In this case, diagnosis of metastatic colon cancer was excluded because there was no glandular structure, and immunohistochemical staining was negative for CK20 and CDX2. Additionally, imaging studies indicated no primary sites of cancerous lesions, including the bladder, breast, salivary glands, and skin. Systemic findings revealed no other primary organs, leading to a diagnosis of primary pleomorphic liposarcoma, epithelioid variant.

Patients with Li-Fraumeni syndrome have a history of multiple malignancies, including a broad spectrum of cancers and soft tissue sarcoma and leukemia. It is caused by germline mutations of the TP53 gene. In this case, patient has a history of multiple malignancies, including colorectal cancer and chondrosarcoma, but no hereditary tumor syndromes, such as Li-Fraumeni syndrome, have been noted.

## 5. Conclusion

Here, we report a case of pleomorphic liposarcoma, epithelioid variant, diffusely positive for GATA3. When pleomorphic liposarcoma shows immunostaining with antibodies against cytokeratin and GATA3, it is challenging to distinguish liposarcoma from metastatic carcinoma, particularly urothelial carcinoma. In cases of sarcoma with epithelioid morphology, the expression of GATA3 should be evaluated carefully.

## Data Availability

All data generated or analysed during this study are included in this published article. Further inquiries can be directed to the corresponding author.
